# Bumblebees negotiate a trade-off between nectar quality and floral biomechanics

**DOI:** 10.1016/j.isci.2023.108071

**Published:** 2023-10-24

**Authors:** Jonathan G. Pattrick, Hamish A. Symington, Walter Federle, Beverley J. Glover

**Affiliations:** 1 Department of Biology, University of Oxford, The John Krebs Field Station, Wytham, Oxford OX2 8QJ, UK; 2 Department of Plant Sciences, University of Cambridge, Downing Street, Cambridge CB2 3EA, UK; 3 Department of Zoology, University of Cambridge, Downing Street, Cambridge CB2 3EJ, UK

**Keywords:** Entomology, Interaction of plants with organisms, Plants

## Abstract

How and why pollinators choose which flowers to visit are fundamental, multifaceted questions in pollination biology, yet most studies of floral traits measure simple relative preferences. Here, we used vertically and horizontally oriented slippery-surfaced artificial flowers to test whether bumblebees could make a trade-off between floral handling difficulty and nectar sucrose concentration. We quantified foraging energetics, thereby resolving the rationale behind the bees’ foraging decisions. The bees chose flowers with either a high handling cost or low sucrose concentration, depending on which was the energetically favorable option. Their behavior agreed with the critical currency being the rate of energy return (net energy collected per unit time), not energetic efficiency (net energy collected per unit energy spent). This suggests that bumblebees prioritize immediate carbohydrate flow to the nest rather than energy gain over the working lifespan of each bee. Trade-off paradigms like these are a powerful approach for quantifying pollinator trait preferences.

## Highlights

• Floral biomechanics influence bee foraging decisions via energetics
• Bumblebees optimized foraging energetics when choosing between floral targets
• Visiting slippery flowers increased energetic expenditure more than handling time
• Our results agree with maximization of rate of energy return, not energy efficiency

## Graphical abstract

**Figure:**
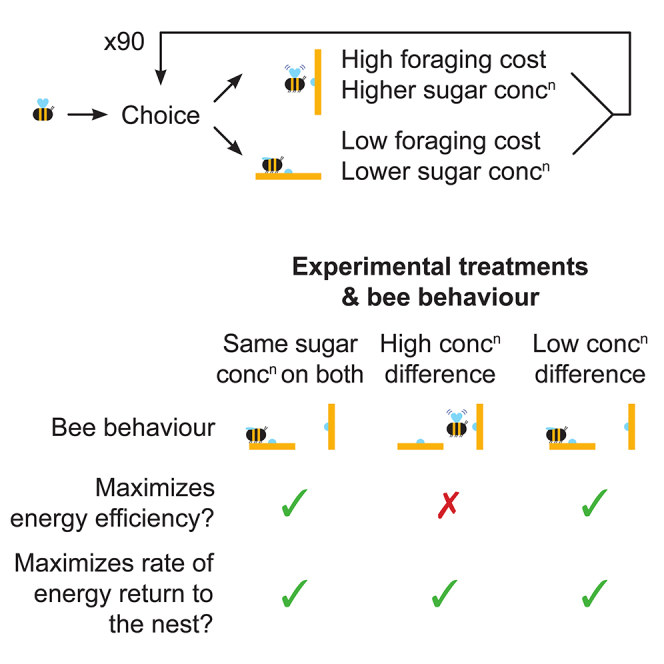

## Introduction

Pollinators typically visit flowers to forage for a nectar or pollen reward. These interactions are shaped by numerous other non-nutritive floral traits such as color,^1^ scent,^2^ and morphology.^3^ A key focus of pollination biology research is in investigating how and why pollinators choose which flowers to visit. Such information is valuable for understanding pollinator-driven evolution of floral traits,^2^^,^^4^^,^^5^ global food security,^6^ elucidating cognitive abilities of animals,^7^ and informing foraging theory.^8^^,^^9^

Much of the work investigating pollinator responses to floral traits involves simple preference assays using a single trait.^1^^,^^3^^,^^10^ While such assays are useful, pollinators in the field are typically faced with flowers from multiple species all differing in reward, and multiple other trait modalities, and in locational and environmental situation, making it difficult to use experimentally determined relative preferences to predict behavior. A trait which is preferred in one context may be avoided in another, for example if the value of the floral reward varies.^11^ One approach to overcome this problem of relative preferences is to quantify the energetic cost/benefit of the trait in question within the overall context of the pollinator’s foraging economics. This gives the ability to make testable predictions about pollinator floral preferences under a wide range of energetic scenarios. Quantification is especially useful for traits that present a biomechanical challenge^3^^,^^12^ to pollinators. This is because a biomechanical challenge for an animal will incur a direct energetic cost, and so is essentially an energetic problem.

Here, we present a trade-off paradigm to explore foraging strategies, trait costs and valuation using the bumblebee *Bombus terrestris*, and the biomechanical challenge of slippery petals,^3^^,^^13^^,^^14^ a trait which alters floral handling difficulty. Our aims were: 1. quantifying the absolute cost of slippery petals to *B. terrestris* and 2. testing whether the bees could adapt their foraging behavior in response to this floral trait in a way which made economic sense. To do this, we presented the bees with a choice between prioritizing floral handling difficulty or nectar reward value.

By quantifying how the choices made by the bees make sense within the overall economics of foraging, we go beyond previous work into foraging trade-offs in bumblebees,^15^^,^^16^^,^^17^^,^^18^^,^^19^ providing a direct energetic rationale for how floral biomechanics influence bee behavior. Achieving this requires identifying absolute trait costs and, the valuation system, (or “currency”) through which the bees are perceiving these costs. Our approach addresses both of these factors, giving the absolute cost of slippery flowers to the bees and allowing us to distinguish between alternative currencies through which a bumblebee might optimize its foraging.

Previous, field-based, studies of nectar-foraging bumblebees have struggled to distinguish between alternative currencies, with the observed behavior of the bees being in agreement with both the currencies of rate of energy return (RER) to the colony (Equation 1) and energy efficiency (EE)^8^^,^^20^^,^^21^^,^^22^^,^^23^ (Equation 2):

(Equation 1) $$RER=\frac{energy\;intake-energy\;expenditure}{time}$$

(Equation 2) $$EE=\frac{energy\;intake-energy\;expenditure}{energy\;expenditure}$$

RER and EE are two key currencies and represent different foraging priorities that both colonies and individual workers could assume. Maximizing RER will maximize the immediate rate of carbohydrate flow to the nest and could for instance be important if colony fitness is limited by the immediate availability of stored resources or by a limited time window for foraging. Alternatively, workers may have a limited foraging lifespan which decreases with increasing energetic expenditure.^9^^,^^24^ In this case, optimizing EE could maximize the lifetime energy return for a worker, even if this implies a reduced instantaneous carbohydrate flow.^25^ Maximizing EE would therefore be important if colony fitness is limited by the cost of rearing new workers.^9^ The currency that bees are seeking to optimize will influence multiple aspects of their foraging behavior such as their nectar sugar concentration preferences,^26^ and so the flowers which they will visit.^27^

Distinguishing between different currencies is also of more general importance. This is because, within the context of foraging, a currency is a criterion for measuring foraging performance which, if correctly identified, provides a link between foraging behavior and fitness. That is, maximizing a currency maximizes fitness.^8^^,^^28^^,^^29^ Consequently, maximizing a currency is likely to be a primary driver of animal foraging behavior.^29^^,^^30^

## Results and discussion

**Figure 1:**
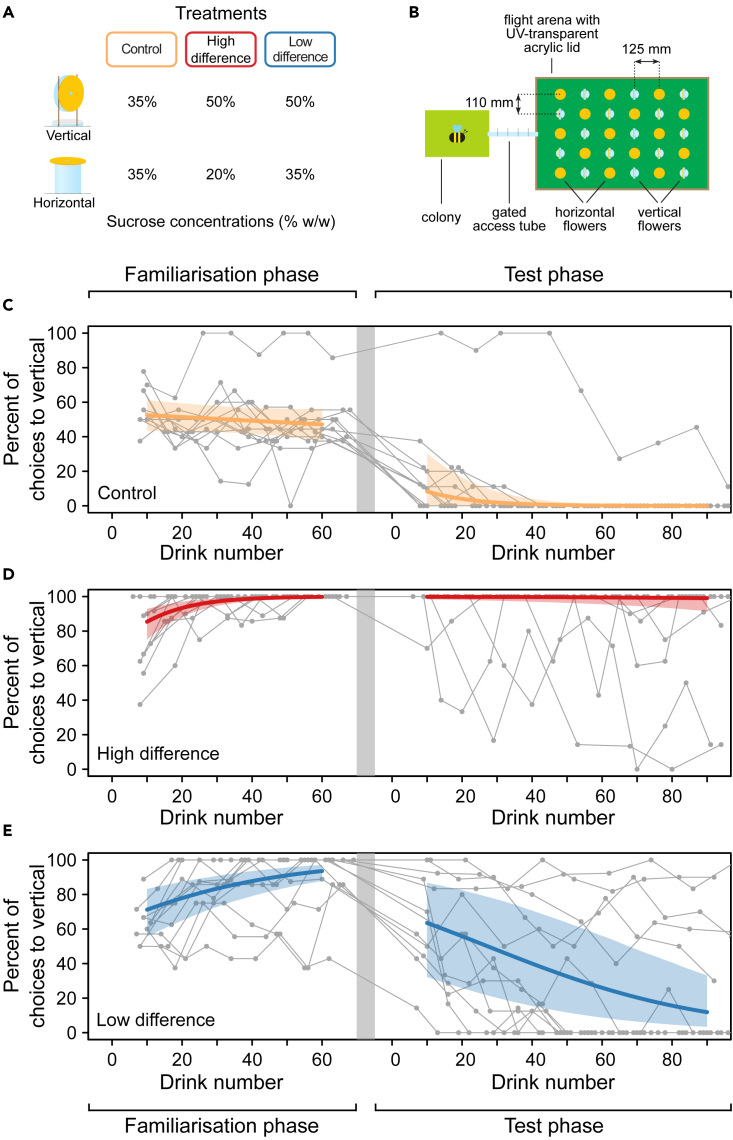
Experimental setup and bumblebee foraging choices (A) Artificial flowers and concentrations of the sucrose solution rewards assigned to vertical and horizontal flowers under the three treatments, see Figure S1 for more details on artificial flower construction. (B) Experimental setup, as viewed from above; vertical and horizontal artificial flowers were arranged in a regular grid in a flight arena connected to a bumblebee colony by a gated access tube. (C–E) The proportion of choices to vertical surfaces for the familiarization and test phases for the control (C), high-difference (D), and low-difference (E) treatments. The measured proportions for each foraging trip for each individual bee are indicated by solid circles, plotted against the cumulative number of choices (drinking visits) made by the end of that foraging trip, with consecutive foraging trips for a bee connected by thin solid gray lines. Thick lines are fitted logistic regression models with 95% CI bounds (shaded area). N = 12 bees per treatment. See Table S2 for fitted model parameters and Figure S2 for behavioral data on all floral visits (i.e., including non-drinking visits).

Worker *B. terrestris* bumblebees (N = 36, 12 from each of three colonies) were individually offered a choice between 15 vertically oriented and 15 horizontal artificial flowers (Figure 1A) (hereafter “flowers”), arranged in a regular array in a lab-based flight arena^3^ (Figure 1B). Floral surfaces were epoxy-resin casts of fine-grit polishing films on which the bees struggled to grip (i.e., they were slippery). Bees feeding from the vertical flowers nearly always had to hover (Video S1) for at least part of their visit (98% of 1,982 drinking attempts). In contrast, bees always landed to forage from the horizontal flowers, and did not need to hover. Forcing the bees to keep beating their wings when visiting vertical flowers increases both their metabolic expenditure and floral handling time.

Video S1. Bumblebee foraging behavior on coarse-rough vertical flowers, used for the familiarisation phase of the experiment, and slippery (micro-rough) vertical flowers, used for the test phase of the experiment, related to Figure 1 Bumblebees could easily grip onto the coarse-rough vertical flowers. In contrast, the bees struggled to grip onto the slippery vertical flowers and typically had to hover to drink the sucrose droplet.

Each flower offered a 15 μL sucrose solution reward, with concentrations (% w/w) within the natural range of floral nectar^27^ and varying according to three treatments (Figure 1A): 1. high difference (vertical flowers = 50%, horizontal flowers = 20%), 2. low difference (vertical = 50%, horizontal = 35%), and 3. control (vertical = 35%, horizontal = 35%). Treatment order was selected at random across bees within each colony, with the constraint of four bees per treatment per colony. The three sucrose concentration treatments were chosen following preliminary work, to create different energetic “trade-off” conditions. The control treatment was included to determine the extent to which bees avoid vertically oriented slippery floral surfaces when the sucrose reward was equal. Calculation of the energetic costs and gains of bees foraging under the different treatments allows testing of whether the bees are able to adjust their foraging behavior in a way which makes economic sense.

Flowers were refilled between each foraging trip. Consequently, there was sufficient sucrose solution for a bee to make rewarded visits solely to flowers of one orientation during each trip. Before testing, bees were familiarized with the floral orientations and sucrose concentrations by using flowers with coarse-rough surfaces which were easy to grip onto (Video S1) until they had completed 60 drinking visits. By the end of this familiarization phase, bees in the high- and low-difference treatments were preferentially drinking from vertical flowers (logistic regression model-predicted mean proportion of choices to vertical (MPMP) [95% CI]: low difference = 93.6% [87.8%, 97.1%]; high difference = 99.8% [99.3%, 100%], Figures 1D and 1E) whereas bees in the control treatment chose vertical and horizontal flowers in equal proportions (MPMP = 47.1% [38.1%, 56.1%], Figure 1C). After familiarization, the coarse-rough flowers were switched for slippery flowers and each bee was observed for 90 drinking visits.

The bumblebees flexibly adapted their foraging to take account of floral surface texture and orientation, with a significant difference in the proportion of choices to vertical flowers between the high- and low-difference treatments by the final ten choices (randomization test, p = 0.00048). When there was a large difference in sucrose concentration between the vertical and horizontal flowers, the bees continued to drink almost exclusively from the vertical flowers (Figure 1D). In contrast, the majority of bees in the low-difference treatment switched to drinking from the horizontal flowers (Figure 1E, MPMP [95% CI] at drink 90: high-difference treatment = 99.1% [91.5%, 100%] low-difference treatment = 11.9% [3.38%, 33.2%]). As illustrated by the bees in the control treatment, which showed a strong aversion to vertical flowers (MPMP [95% CI] at drink 90 = 0.02% [0.00%, 0.12%], Figure 1C), bumblebees usually avoid slippery flowers in situations when good attachment is important.^3^^,^^13^ Here, we demonstrate that the bees can override this aversion, but only if the reward is worth it, implying that they are making an economic decision in their foraging behavior.

To explore the economics of the trade-off, we used a custom behavioral logging program which mapped keystrokes to different behavioral activities, adding a timestamp for each. This permitted recording of the sequence of behavioral activities and the time each bee spent on each distinct activity during each foraging trip. Using this information and our data from a previous study on drinking rates,^26^ we could produce reliable estimates of the volume of sucrose solution consumed (Figure S3), hence giving energetic gain for each foraging trip. In line with other work determining metabolic costs of bumblebee foraging,^30^^,^^31^^,^^32^ energetic expenditure was estimated using published values for bumblebee metabolic rate.^26^^,^^33^^,^^34^ There is good agreement on metabolic rate for bumblebee flight among studies^20^^,^^31^^,^^33^^,^^35^ and our conclusions are robust to variation in the values used (see supplementary information). We calculated the energetics of each foraging trip according to two energy currencies: the RER to the colony in J s^−1^ (Equation 1) and EE (dimensionless, Equation 2).^8^^,^^9^

For both the high- and low-difference treatment, we also calculated estimated energetic parameters for RER and EE under the assumption that bees use the alternative strategy to that observed: that is, if the bees in the high-difference treatment had switched to mostly foraging on horizontal flowers (calculated using behavioral data from the control bees) and if the bees in low-difference treatment had mostly continued foraging on the vertical flowers (calculated using the behavioral data from the high-difference bees). This allows for a comparison of the energetics of foraging on vertical vs. horizontal flowers within the different treatments.

**Figure 2:**
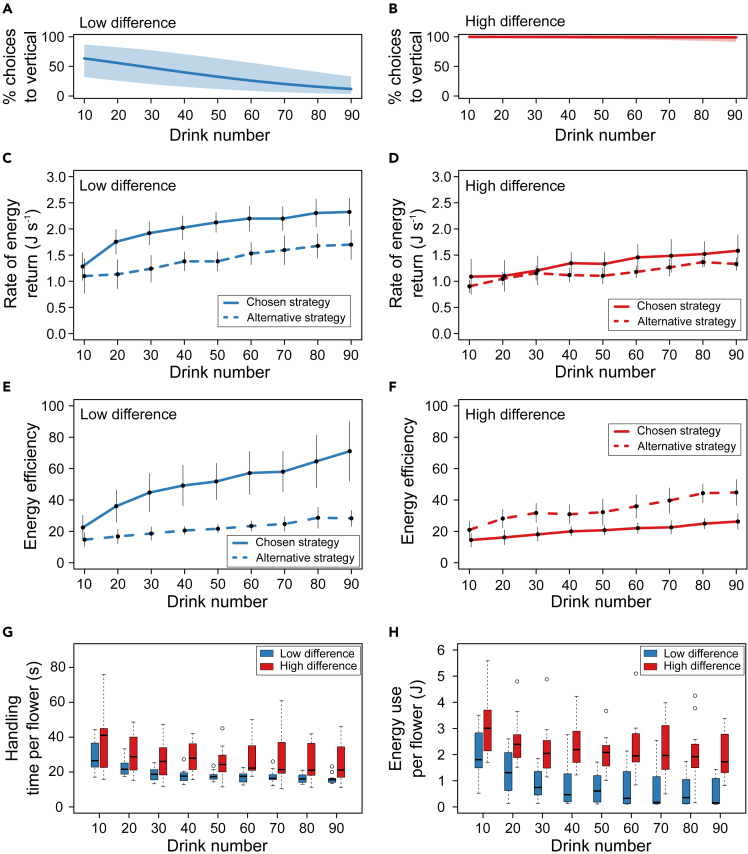
Foraging energetics on slippery flowers (test phase) (A and B) Summary (fitted logistic regression models ±95% CI bounds) of foraging choices to slippery vertical flowers (test phase) for the low-difference (A) and high-difference (B) treatments. (C) Mean RER to the colony at every ten choices for the low-difference treatment. The solid line is the observed behavior, the dashed line is the estimate if the bees had instead chosen the alternative strategy (predicted from behavioral data from the high-difference treatment, see STAR methods). (D) As in C, but for the high-difference treatment (values for the alternative strategy are predicted using behavioral data from the control treatment, see STAR methods). (E) As in C, but plotting mean EE every ten choices. (F) As in D, but plotting mean EE every ten choices. In (C–F), error bars are 95% CIs; points are slightly offset for visibility. (G) Boxplots of handling times per floral choice, calculated as the mean of any time spent interacting with a flower over every ten choices. (H) Boxplots of energy expenditure per floral choice, calculated as the mean of the energy expended during any time spent handling flowers over every ten choices. In all panels, N = 12 bees per treatment. See Figure S4 for foraging energetics during the familiarization phase and for the control treatment. All boxplots show the median (thick horizontal line), and an interquartile range (hinges). The whiskers extend in each direction to the most extreme data point which is no more than 1.5x the interquartile range away from the hinges. Unfilled circles represent outliers.

Our results agree with expectations if the bees maximize RER while foraging. In the low-difference treatment, there was a clear energetic benefit to the bees in switching to the horizontal surfaces (Figure 2C). For their first ten choices, the bees in the low-difference treatment were still drinking from the vertical flowers (MPMP = 63.5% [32.3%, 86.7%]) and had an RER not significantly different from the high-difference-treatment bees (Tukey, q_3.33_ = 1.37, p = 0.60). However, as the bees switched to drinking mainly from the horizontal flowers, the RER increased such that, by the final ten choices, the RER was significantly higher than estimated if the bees had continued to forage from the vertical flowers (t test, t_21.88_ = 3.16, p = 0.0045, Figure 2C).

Despite the fact that bees in the high-difference treatment mainly foraged from vertical flowers with the higher-value reward, they had a significantly lower RER over the final ten choices than the bees in the low-difference treatment (Figures 2C and 2D, Tukey q_3.33_ = 5.48, p = 0.0014). The RER for the alternative strategy available to the high-difference-treatment bees (switching to foraging from horizontal flowers with 20% sucrose, and predicted using foraging behavior of the bees in the control treatment) was not significantly different from the RER of their observed behavior of foraging from vertical flowers (t test, t_14.46_ = 1.52, p = 0.15, Figure 2D). Consequently, there would be no benefit (in terms of RER) for these bees in switching. This constancy to vertical flowers agrees with previous work showing that, after an effective downshift in reward, bumblebees continue foraging from familiar flowers if energetic outcomes are equal across the available options.^36^

In contrast to the results for RER, the combined behavior of the bees across treatments was *not* consistent with maximizing EE. Bees in both high- and low-difference treatments could have attained a higher EE by switching to horizontal flowers, but only the low-difference-treatment bees actually switched. For the first ten choices, EE was similar for bees in the low- and high-difference treatments (Tukey, q_3.33_ = 2.92, p = 0.11, Figures 2E and 2F). By the final ten choices, EE for the low-difference-treatment bees was over 2.5 times higher than estimated if the bees had continued foraging on the vertical flowers (t test, t_18.41_ = 4.66, p = 0.00018, Figure 2E). For bees in the high-difference treatment, which mainly foraged from vertical flowers, EE for the final ten choices was significantly lower (t test, t_18.05_ = 3.81, p = 0.0013) than that predicted (using data from the bees in the control treatment) if the bees had switched to the horizontal flowers (Figure 2F). Consequently, our results suggest that the bumblebees are maximizing the currency of RER rather than maximizing EE.

An explanation for why foraging on slippery flowers impacts EE more than RER is provided by examining the breakdown of foraging costs for the final ten choices. RER and EE both increased over the 90 choices (Figures 2C–2F, S4A, and S4B); however, comparison across treatments and between the experimental test and familiarization phase suggests that by the final ten choices for both currencies, values were at or approaching asymptotes (Figures S4C–S4E, supplementary information). The handling time over the final ten drinking visits for bees in the high-difference treatment (median = 21.2 s), which were mainly foraging from vertical flowers, was about 1.3 times higher than that of the low-difference-treatment bees (median = 15.6 s) (t test, t_13.76_ = 2.72, p = 0.017, Figure 2G) which were mainly foraging from horizontal flowers. In contrast, the median energy used by the bees per foraging visit was 10.9 times higher in the high-difference treatment than the low-difference treatment (t test, t_14.33_ = 5.09, p = 0.00015, Figure 2H). This was due to the high energetic cost of hovering when visiting the vertical flowers. RER is much more dependent on the time taken than the energy used, whereas EE is dependent on the energy used, but not directly on time. The overall cost of the slippery flowers can be estimated by comparing bees in the control and high-difference treatments. This gives an increase in the median time taken per successful visit from 14.3 to 21.2 s and an increase in the median energy used from 0.13 to 1.72 J.

Our work highlights how floral traits which act as filters^12^ do not necessarily restrict access to flowers in a binary manner. Instead, whether or not the cost imposed by that trait stops the pollinator visiting is dependent on the situation, with bumblebees overriding their preference for flowers that are easy to land on if it is economically favorable for them to do so. Even in the context of bumblebees’ impressive cognitive abilities,^7^ the behavioral flexibility shown here is remarkable. The bees in the low-difference treatment mostly chose 35% sucrose over 50% sucrose. This is notable given that, excepting situations involving avoidance of predation,^18^^,^^19^ there is overwhelming evidence from previous work that at this stimulus intensity,^37^ the preference should be for the higher concentration^27^^,^^38^ (Figures 1D and 1E). Sucrose concentration preferences in bumblebees are clearly not fixed, but flexible such that they can be integrated with other parameters in order to optimize foraging economics.

Foraging optimally is critical for the fitness and success of bumblebee colonies as they only have a limited time window in which to grow and reproduce.^39^ Our identification that RER is the currency used by nectar-foraging bumblebees provides a framework for understanding how foraging choices influence colony success and pollination efficiency. Maximization of RER suggests that bumblebees will prioritize the immediate rate of energy flow to the colony over worker longevity and that their foraging choices will be more heavily influenced by factors that affect the duration of foraging than those which affect the energetic cost of foraging. Our work goes beyond previous modeling approaches based on field observations, which could not distinguish between the alternative currencies of RER and EE.^8^^,^^20^^,^^21^^,^^22^^,^^23^ Combining our results on trait costs with the currency of RER also facilitates prediction of bumblebee preference for slippery flowers in a wide range of situations. Furthermore, we suggest that our experimental paradigm has significant value in calculating how other floral traits influence reward evaluation by bumblebees.

Differences in nectar-foraging currencies will likely contribute to between-species differences in floral choices. For example, here we found that the cost of choosing vertical slippery flowers is much larger in terms of increasing metabolic expenditure than increasing foraging time. Bees which are seeking to maximize EE should therefore value such flowers as comparatively more expensive to visit than bees which maximize RER. Notably, honeybees appear to maximize EE rather than RER.^9^^,^^40^^,^^41^^,^^42^^,^^43^ Honeybees are more reliant on information from colony members in their foraging decisions^44^ and this, or the differing colony structure and lifecycles from bumblebees,^8^ could explain the contrasting foraging strategies. There has been less work on foraging currencies in solitary bee species; however, they are likely to face different selective pressures than workers of social bee species. Unless directly provisioning a nest, in which case maximizing either RER or EE may be appropriate currency choices, solitary bees will be foraging to feed themselves and so maximizing energetic uptake while on a flower^45^ may be of chief concern. Additionally, injury or death is much more costly in terms of inclusive fitness to a solitary bee than a social bee worker and so minimizing predation risk^42^ is likely to be of high importance. Males^46^ and newly emerged queens of social bee species may be in a similar situation.

More widely, experimental studies using trade-offs, particularly between time to reward and reward value, are frequently used to study decision-making in diverse animal species, including birds, rats, and humans.^47^^,^^48^^,^^49^ Such work explores the extent to which animals can display self-control through delayed gratification trials^47^^,^^48^ and, through delay discounting frameworks, how the values of future rewards are discounted according to the time delay until they are received.^49^ There is an obvious advantage to studying such paradigms in bumblebees, namely the ability to directly quantify costs and benefits of alternative strategies. Rather than using relative measures of reward value,^48^ quantification of foraging economics can be used to ask more targeted questions, both specifically with regards to pollinator behavior and also broadly across foraging theory.

### Limitations of the study

Here, the behavior of nectar-foraging bumblebees is in agreement with maximizing RER but not EE. This conclusion relies in particular on the behavior of the 12 bees in the high-difference treatment and so, given this moderate sample size, some caution should be applied to our findings. There was also variability in bee behavior. Notably, two of the bees in the high-difference treatment switched to mainly foraging on horizontal flowers (Figure 1D); however, there was no evidence that the remaining 10 bees were in the process of switching to foraging from horizontal flowers (Figures 1D, S2, and Table S2). Our results are from bumblebees foraging under one experimental paradigm only, and it is possible that the optimal or chosen currency depends on the specific circumstances.^25^^,^^29^ Over longer timescales, animals may use more complex currencies than RER or EE.^29^ Future work should aim to test if RER is consistently used by bumblebees, in particular under other foraging conditions and colony states (for example, if the colony has abundant stored nectar^25^).

## STAR★Methods

### Key resources table

**Table:** 

REAGENT or RESOURCE	SOURCE	IDENTIFIER
**Deposited data**		

Bee observation data (CSV)	This paper	https://doi.org/10.17863/CAM.100572

**Experimental models: Organisms/strains**		

*Bombus terrestris audax*	Agralan, Ashton Keynes, UK	N/A

**Software and algorithms**		

R (4.1.3)	The R Foundation	N/A
R lme4 1.1–28	(Bates et al. 2015)	N/A

**Chemicals, peptides and recombinant proteins**		

Aluminum oxide polishing films	Ultratec, Santa Ana, CA, USA	N/A
Sylgard 184 silicone elastomer (polydimethylsiloxane)	Dow Corning, Barry, UK	N/A
Elite HD + light body, normal set (polyvinyl siloxane)	Zhermack, Badia Polesine, Italy	N/A
Epoxy resin PX672H	Robnor Resinlab, Swindon, UK	N/A
Yellow pigment 586522	AMI, Kaltenkirchen, Germany	N/A
White pigment (zinc white)	L. Cornelissen & Son, London	N/A

### Resource availability

#### Lead contact

Further information and requests for resources should be directed to and will be fulfilled by the lead contact, Jonathan Pattrick (jonathan.pattrick@biology.ox.ac.uk).

#### Materials availability

This study did not generate new unique reagents or materials.

### Experimental model and study participant details

#### Bumblebee foraging setup

*Bombus terrestris audax* bees were obtained from Biobest (supplied by Agralan, Ashton Keynes, UK) and housed in plastic nest boxes of approximate size 292 × 225 × 240 (L x W x H) mm inside a close-fitting cardboard box. Nest boxes were connected via a gated transparent tube to a 0.3 × 0.75 × 1.12 m (H x W x L) flight arena constructed from wood with a clear UV-transparent acrylic lid (Figure 1B). The gates in the connecting tube could be used to allow specific bees to enter and leave the arena and restrict access to a single focal bee during experimental trials. The base of the arena was painted green with Plasti-Kote fast-drying enamel, B9 “Garden Green”. The flight arena was illuminated from above with Sylvania professional Activa 172 58 W fluorescent tubes and additional lighting was provided by a desk lamp with an Ecozone 25 W daylight bulb (Ecozone, London, UK).

Bees were marked on the dorsal side of the thorax with numbered tags (queen marking kit from Abelo, Full Sutton, York, UK) attached with superglue (Gorilla Glue, Chorley, UK). Tags + glue weighed approximately 3 mg, which is around 2% of the mass of bumblebee foragers. Marking with tags was carried out with a queen marking cage (Thorne, Rand, UK). The dorsal surface of the thorax of the bee was shaved with a razor blade prior to tagging to ensure better adhesion of tags and enable easy discrimination between newly emerged bees and those which had lost tags. Bees were not used for experimental tests on the day they were tagged.

When colonies were not directly in use for experiments, they were fed with approximately 20% w/w sucrose solution in feeders of 96-well PCR plates (Starlabs, E1403-0100) which were placed in the flight arena. Colonies were typically supplied with pollen *ad libitum* directly to the colony in the nest box.

Bees were selected for experiments by choosing motivated workers. Worker bees in this setup generally collect sucrose solution in discrete foraging trips. They leave the colony, collect sucrose solution from the feeders and then return to the colony to offload the sucrose solution. Motivated workers were defined as those observed to complete several consecutive successful foraging trips. Worker bees were tested individually.

### Method details

#### Preparation of substrates for artificial flowers

Circular replica surfaces of aluminum oxide polishing films (Ultratec, Santa Ana, CA, USA) (hereafter referred to as ‘discs’) were made for use as foraging surfaces. Films with nominal particle sizes of 12 μm were used to make the slippery (micro-rough) discs and films with particle sizes of 30 μm were used to make the coarse-rough discs. Disc particle size and diameter were selected following preliminary experiments, which showed that bumblebees had difficulty gripping onto discs cast from 12 μm polishing film, but could easily grip onto discs cast from 30 μm film. Additionally, when the diameter of the discs was too small, a bee could easily grip onto the edge of the disc and extend its proboscis to feed from a droplet of sucrose solution at the disc’s center, thus circumventing the need to grip onto the disc surface itself. Discs were therefore made to be of a sufficiently large diameter (58 mm) to counter this problem.

Imprinting techniques using silicone-based polymers have been shown to give highly accurate replicas of natural and artificial surfaces.^50^^,^^51^ They have also previously been used as negative molds for epoxy resin surfaces for creating surface replicas for behavioral experiments with bumblebees.^3^

Polydimethylsiloxane (PDMS) molds were prepared using a PDMS mix with a setting time of several hours, which allowed ample time for removal of air bubbles which can disrupt the integrity of the cast surface. PDMS negative molds were created in batches of four. Four 58 mm-diameter circular sections of the chosen polishing film surfaces were glued using epoxy resin (2 Ton epoxy (Devcon, Danvers, MA, USA)) inside the base of a square Petri dish (120 × 120 mm) in a square grid pattern. PDMS base and curing agent (Sylgard 184 silicone elastomer, Dow Corning) were mixed in a 10:1 ratio by mass and degassed for approximately 25 min in a vacuum chamber (circa −0.9 bar negative pressure) to remove all bubbles, and around 45 g was added to the square petri dishes with polishing film templates. The filled templates were allowed to set for 72 h and then cut out of the Petri dishes. To complete the mold, a polyvinyl siloxane (dental impression mold Elite HD + light body, normal set, Zhermack) retaining wall was formed around each set of four PDMS negative impressions.

The discs were created from the molds using epoxy resin (PX672H, Robnor Resinlab, Swindon, UK). Yellow (Künstler-Pigmente 586522, AMI, Germany) and white (zinc white, L. Cornelissen & Son, London) powdered pigments were added to the resin pre-polymer and mixed thoroughly, giving an opaque yellow color. Pigments were added so that final concentrations (percentage mass) in the complete epoxy resin were 3.79% and 1.88% for the yellow and white pigments respectively. Resin (with pigment) and hardener were mixed in 2.4:1 ratio by mass (adjusting for the mass of pigment) and degassed in a vacuum chamber (as above) for approximately 8 min. Visible bubbles were then drawn from the surface of the mixture with a plastic Pasteur pipette and 7 g of the pre-polymer mixture was added to each mold. Filled molds were degassed for circa 20 min and then allowed to set for a minimum of 24 h.

#### Artificial flowers

The discs (all yellow) were used to create artificial flowers, oriented horizontally or vertically (Figures 1B and S1). For the vertical flowers, discs were attached using adhesive hook and loop fastening tape to a support stand consisting of a 58 mm Petri dish lid glued with epoxy resin to two pieces of 3 mm bamboo dowelling (length approx. 127 mm), held vertically in a Hamilton jar (60 × 39 mm, height x diameter) using a polyurethane foam bung and superglue. For the horizontal flowers, discs were placed on taller Hamilton jars (69 × 34 mm, height x diameter). The height of both supports was such that the surfaces of the flowers in the horizontal orientation were level with the bottom of the surfaces of flowers in the vertical orientation. The Hamilton jar for horizontal flowers also contained a foam bung and a small quantity of epoxy resin and superglue to control for any effects of odor (Figure S1); jars for both horizontal and vertical supports were covered with Parafilm (Bemis, Sheboygan Falls, Wisconsin, USA).

The experiment required that a drop of sucrose solution could be added to the center of a disc such that if the disc was oriented vertically (i.e., perpendicular to the ground), a bee had to either land and grip on the disc surface or hover in front of it to forage from the disc. Preliminary trials showed that 15 μL droplets of sucrose solution stayed in place on vertical surfaces by surface tension.

#### Training

Worker bees, naive to the yellow discs/artificial flowers, were trained to associate the yellow discs with a sucrose solution reward over five to six foraging trips using two artificial flowers, also of yellow epoxy resin discs, created in a similar fashion to the test discs, but with very rough surfaces (replicas of 180 grit sandpaper). The surface of these flowers was oriented at 45° to the horizontal in an effort to avoid imparting any bias toward horizontally or vertically oriented flowers before the actual experiment. Bees from colony 1 were subjected to a sixth training trip, with 15 μL droplets presented on one horizontal and one vertical artificial flower; each flower was replenished when the bee was drinking from the other. Depending on the treatment, one or two different sucrose solution concentrations were used in the actual experiment (see below), therefore during training with each bee, the sucrose concentration used was always the lower concentration that the bee would encounter during the actual experiment. Given this, during training, the sucrose concentration was 35% (% w/w) for the control and low-difference treatments and 20% for the high-difference treatment.

#### Experimental setup

After training, bees were tested individually using a setup with 30 yellow flowers, 15 oriented vertically, 15 horizontally, in a rectangular grid of 6 columns and 5 rows (Figure 1B). There was 125 mm between each column and 110 mm between each row, center to center. Flowers were arranged so that they alternated between horizontal and vertical along both rows and columns. Before the start of each foraging trip, a 15 μL droplet of sucrose solution was placed in the center of each flower. Three different sets of sucrose (table sugar, Tate and Lyle, UK) concentrations (% w/w) were used, depending on the treatment: 1. High difference – 20% for the horizontal flowers, 50% for the vertical flowers; 2. Low difference – 35% for horizontal flowers, 50% for vertical flowers; 3. Control (no difference) – 35% for both horizontal and vertical flowers. Concentrations were selected following preliminary work to identify under which conditions bees continued foraging on the vertical flowers and when they switched to foraging on the horizontal flowers. Although flowers were not refilled during a foraging trip, the use of 15 flowers per floral orientation with 15 μL of solution per flower (i.e., 225 μL per orientation per trip) ensured that a bee could fill up solely on flowers of one orientation.

The experiment was split into two parts: a familiarisation phase and a test phase. In the first part (the familiarisation phase), all flowers had a coarse-rough (30 μm replica) surface, and the bee was allowed to forage until the end of the foraging trip in which she completed her 60th drinking visit (see below). This gave time for the bee to taste the sucrose concentration on both flower orientations and learn and display any preference for orientation or concentration, thus testing for any effect of orientation without any difference in surface texture. The setup was then changed for the second part (the test phase) so that all flowers had a slippery (micro-rough) surface (replica of the polishing film with 12 μm particle size). The bee was allowed to forage until the end of the foraging trip in which she completed her 90th drinking visit. The familiarisation and test phases took on average 4.4h ±0.9 (mean ± sd) per bee, with a minimum of 2.6 h and a maximum of 6.8 h. Additionally, training took approximately 1–1.5 h. For each bee, after training had begun, that bee was taken through training, the familiarisation and test phases before moving on to the next bee. For any given bee, all training and experimental trials were completed within one day.

The experiment was performed with twelve bees for each treatment, selected from three colonies. Four bees were used per treatment per colony. The order of treatment assignment was random within each colony. In addition to the 36 bees analyzed for the experiment, two bees were trained and finished the familiarisation phase but did not complete the experimental test.

During each foraging trip, a simple custom computer program written in Xojo v2017r1.1 and compiled for macOS X was used to record the orientation of flowers which the bee visited and drank sucrose solution from, as well as the time spent visiting, drinking, flying between flowers, or resting. Recordings were triggered in real-time using keyboard shortcuts. This wrapped Structured Query Language (SQL) statements to different keystrokes, with a different keystroke for each behavioral activity. Alongside each observation/behavior a timestamp in milliseconds was recorded, giving a sequence of such behavioral observations for each bee. With practice, the software could be used by touch alone so that the researcher could maintain eye contact with the test bee. Observations were all made by a single researcher, who used the software for over 20 h during refining of the experimental protocol, and who was therefore well practised in its use.

As we were primarily interested in the flowers from which the bee chose to actually consume the sucrose solution, a “choice” was defined as a bee drinking from the sucrose solution droplet on a flower. Drinking was defined as the bee contacting the sucrose solution with her tongue (glossa) for over 2 s. This 2 s cut-off was chosen to exclude instances where the bee tasted but did not drink from a droplet. We also recorded all visits to flowers (whether or not the bee drank from the flower). Visits (whether drinking or not) initially reflect any innate preference of the bee for horizontal or vertical flowers; visitation rates to horizontal or vertical flowers will subsequently change as the bee learns to associate the combination of sucrose concentration and handling difficulty of that flower with its orientation. Contrastingly, drinking visits are a more immediate reflection of the sucrose concentration preference and handling difficulty of the flowers. If the bee left a flower then returned to the same flower within 10 s without having visited any other flower, the return visit was counted as part of the original visit.

While bees were drinking from and/or visiting vertical flowers, we recorded whether or not the bee landed on the flower (defined as having her wings still) or whether the bee was hovering (defined as having her wings moving). Hovering therefore included where the bee was unable to land but attempted to do so, scrabbling at or slipping on the flower/disc surface. The duration of any periods of hovering versus landing were recorded. ‘Hover visits’ only occurred on vertical flowers. When a bee made several contacts with the solution (as frequently happened in hover visits, where the bee could not grip onto a slippery flower), all drinking durations were summed for that visit, with the visit being defined as a drinking visit (i.e., a “choice”) if the sum exceeded 2 s. If a bee tasted the sucrose solution and then did not continue to forage (tongue in sucrose solution for 2 s or less) this was not recorded as a choice.

Particularly during hover visits, a bee would sometimes not drink all of the sucrose solution from a flower during a single visit, and could subsequently drink from the same flower one or more times during a foraging trip.

#### Resetting the artificial flowers between trips

Between trips, discs were washed under running cold water and patted dry with paper towel. Bumblebees can use scent marks deposited by bees of the same and other species to avoid previously visited (i.e., potentially unrewarding) flowers.^52^^,^^53^ In this experiment, it was not possible to remove scent marks with the standard procedure of wiping with ethanol^3^ as preliminary work showed that repeated wiping of disc surfaces with ethanol degraded the fine microstructure. To control for this, between each foraging trip and before assignment to a horizontal or vertical flower, discs were shuffled, such that orientation and placement (horizontal/vertical and grid position) was essentially randomized. Positions of vertical and horizontal supports were alternated between foraging trips. Between bees, to aid in dissipating volatiles, discs were held at 37°C for at least 2 h, and supports were wiped with 70% ethanol. Before the beginning of the next foraging trip, flowers were refilled with 15 μL of the appropriate sucrose solution.

#### Measuring bee mass after the experiment

After the end of the test phase, the bee was permitted to return to the colony to deposit the contents of her honey stomach in a honey pot. Upon emergence she was captured, euthanised by freezing and weighed. Foragers that have already made multiple foraging trips emerge from the colony minimally laden.^26^ One bee (Pink 60, Colony 1) failed to re-emerge from the colony after the test, and it was not possible to retrieve her (living or dead) from inside the colony thereafter. We estimated her mass using the mean mass of the remaining bees (171 mg).

#### Estimation of volume of sucrose consumed

We have previously measured drinking speed of *B. terrestris* on 35% and 50% w/w sucrose.^26^ Using the raw data from that experiment, we re-modelled the relationship between log_10_(drinking rate) and log_10_(bee mass) as linear models. We constructed separate models for 35% and 50% sucrose, generating power law equations of the form

(Equation 3) $$\frac{volume\;consumed}{drinking\;time}=a\times {\left(\frac{bee\;mass}{m_{1}}\right)}^{b}$$

where *a* is in μl s^−1^, *b* is dimensionless, the constant *m*_*1*_ = 1 g, volume is in μl, drinking time is in seconds, and bee mass is in g.

For 50% sucrose, *a* = 1.542 μL s^−1^, and *b* = 0.155. For 35% sucrose, *a* = 2.991 μL s^−1^ and *b* = 0.4602. Previous work has found that drinking rate is constant below around 35–40% w/w sucrose^54^ and so we used the same model for 20% sucrose as for 35% sucrose. We made the assumption that drinking rate (when the bee’s proboscis was in contact with the sucrose solution) was the same on vertical and horizontal flowers and while landed and hovering. Our models gave good estimates of the volume consumed. On surfaces where the bees were easily able to forage (all horizontal surfaces and coarse-rough (30 μm) vertical surfaces), we observed that the bees typically drank all of the 15 μL solution per flower, if the flower was their preferred choice out of the two options available. Accordingly, on these treatment/surface/orientation combinations, histograms of the estimated volume consumed per flower all have peaks at, or very close to, 15 μL (Figure S3). Also apparent from these histograms by the high frequency of low volume estimates is where a flower type was not preferred. Bees had difficulty drinking from the slippery vertical surfaces, and so there were few drinking visits with an estimated volume consumed of 15 μL, even in the high-difference treatment, where this flower type was preferred by the majority of bees. Due to the variation of drinking rate, it is to be expected that our models would lead to some estimates of a drinking volume >15 μL (Figure S3). Given that 15 μL was the upper limit of the volume of solution a bee could consume per flower, any estimates >15 μL were set to 15 μL before being used for the calculation of foraging energetics. Any estimates of volume consumed that were equivalent to a drinking time of 2 s or less (see above) were not included in total drinking volume, as preliminary observations suggested bees were typically tasting, but not drinking, the solution.

#### Calculation of foraging energetics

We calculated foraging energetics according to two currencies^8^^,^^9^: rate of energy return to the colony (RER) (Equation 4) and energy efficiency (EE) (i.e., the ratio of energy gained to energy used) (Equation 5).

(Equation 4) $$RER=\frac{energy\;intake-energy\;expenditure}{time}$$

(Equation 5) $$EE=\frac{energy\;intake-energy\;expenditure}{energy\;expenditure}$$

Both RER and EE were calculated per foraging trip. Energy intake was calculated using the estimated volume of sucrose solution consumed on each flower visit. The concentration-specific density (ρ_c_, in g mL^−1^) of the sucrose solution was obtained following Prŷs-Jones & Corbet,^55^

(Equation 6) $$\rho_{c}=0.9988603+0.0037291c+0.0000178\;c^{2}$$

where *c* is the sucrose concentration (in % w/w). The mass of sucrose consumed per flower was calculated by multiplying this density by the volume and the sucrose concentration (c/100):

(Equation 7) $$mass\;of\;sucrose=volume\times \rho_{c}\times \frac{c}{100}$$

where mass is in g and volume in mL.

Total sucrose intake from all drinking visits was summed across each foraging trip; energy intake was obtained by multiplying this by the energy content of sucrose (15.48 J mg^−1^).^54^ Energy expenditure was calculated using the time the bee spent on each behavioral activity (e.g., flying/probing flowers/resting) across a foraging trip and the mass-specific metabolic rates for a bee in flight (0.435 J g^−1^ s^−1^) and while probing a flower (0.034 J g^−1^ s^−1^).^20^^,^^26^^,^^33^^,^^34^^,^^54^ The mass-specific metabolic rate for flight was used when the bee was flying between flowers or visiting/drinking from a flower while hovering. The mass-specific rate for probing was used when the bee was resting, or visiting/drinking from a flower while landed. At the start of a foraging trip we used unladen bee mass (see above) however, the mass of the bee was updated over the course of the trip as the bee consumed sucrose solution, such that the metabolic rate increased over a foraging trip. It has been shown that the metabolic rate of a bee while loaded can increase much less than expected using this simple model^35^; however, robustness testing of our models found that even using the extreme condition of no increase in metabolic rate with load, there was little impact on the energetic parameters and no effect on our conclusions.

After offloading, bees sometimes emerged from the colony before the experiment had been reset. In this case, they were held in the tube until the arena had been prepared for the next foraging trip. We did not include this time in calculation of energetics, making the assumption that this would not be included in the bee’s assessment of their foraging performance; however, we did include time for offloading sucrose solution and for any other time spent in the nest (e.g., searching for a honeypot to offload into). Offloading time was estimated using models from our previous work,^26^ such that

(Equation 8) $$Offloading\;time=10^{-1.652}\frac{s}{\text{μl}}\times {\left(\frac{\mu_{c}}{1\;\mathrm{mPa}\;s}\right)}^{0.502}\times volume$$

where $\mu_{C}$ is the viscosity of the solution at concentration *c*, calculated using the Génotelle equation,^56^ with the same coefficients as Pattrick et al.,^26^ and assuming the abdominal temperature of the bee (in °C) was 16.8+0.438×labtemperature,^57^ where lab temperature was measured as 21°C. Other time spent in the nest was fixed at 83.8 s, the mean time spent in the nest (excepting time spent offloading solution) recorded from 30 bumblebees.^26^ The mass-specific metabolic rate for probing was used for calculating energy expenditure for all time spent in the nest.

#### Estimating the cost of slippery vertical flowers

To estimate the cost of foraging on slippery vertical flowers over horizontal flowers in terms of time and energy expenditure we used data from the final ten drinking visits during the test phase for the bees in the control and high-difference treatments. The bees in the control treatment were almost exclusively foraging on horizontal flowers at this time, and so give a good estimation of the cost of foraging on horizontal flowers. The bees in the high-difference treatment were mostly foraging on vertical flowers and so give a good estimation of the cost of foraging on slippery vertical flowers. Time spent was calculated using the median time spent per drinking visit on any interaction with flowers (i.e., both drinking visits and visits) over the final ten drinking visits for each treatment. Energy expenditure was calculated using the median energy used per drinking visit (as detailed above) during any interaction with flowers (i.e., both drinking visits and visits) over the final ten drinking visits for each treatment.

### Quantification and statistical analysis

All statistical analyses were performed using R version 4.1.3^58^ with a sample size of n = 36 bees, 12 per treatment. An additional two bees were trained and finished the familiarisation phase but did not complete the experimental test and so were not included in the analyses. The proportion of drinks and visits to vertical flowers across the familiarisation and test phases were each modeled using generalised linear mixed models with a binomial error structure using the R package lme4, version 1.1–28.^59^ For these models we calculated the respective proportions across each foraging trip for each bee, using this as the response variable. The total number of completed drinks since the start of the experiment by the end of the respective foraging trip was used as the predictor. Bee ID was entered as a random factor (intercept) in these models. Model fits were examined using the DHARMa package, version 0.4.5.^60^ The proportions and associated 95% confidence intervals reported (Figures 1C–1E, results, Table S2) were calculated using model predictions. The confidence intervals were calculated using a bootstrap method (bootMER function in lme4, with re.form = NA) with 10,000 simulations. For comparing the proportion of the final ten choices which were to vertical flowers between the high and low-difference we used a randomisation test,^61^^,^^62^ using the mean difference in proportions between treatment groups as the test statistic and a null distribution of 100,000 resampled values (see results).

RER and EE were calculated per foraging trip. Although there was no effect of treatment on the number of drinking visits per trip (Table S1), bees took differing numbers of foraging trips to complete the 90 drinking visits of the test phase. Therefore, to facilitate statistical comparisons between bees as they progressed through the test phase of the experiment, we calculated a value for RER and EE for every group of ten drinks. Firstly each behavioral event was assigned a value for RER or EE equal to that for the foraging trip on which that event occurred. The mean RER and EE were calculated across behavioral events for every drinking visit and then for every ten drinking visits (±95% CI, Figures 2C–2F, S4A and S4B). Averaging this way ensured that where behavioral events between two consecutive drinking visits or a group of ten drinking visits included the transition between two foraging trips, the average RER/EE was weighted appropriately based on the RER/EE of the two trips.

Bees in the low-difference treatment mostly switched to foraging on the horizontal flowers, whereas bees in the high-difference treatment mostly continued foraging on the vertical flowers. Using our data we estimated the RER and EE for the alternative behavioral strategy (i.e., if bees in the low-difference treatment had continued foraging on the vertical flowers, or if the bees in the high-difference treatment had switched to foraging on the horizontal flowers). To calculate estimated energetic parameters (means ± 95% CI) for the bees in the high-difference treatment assuming that they had switched to foraging on the horizontal flowers (Figures 2D and 2F), we used the behavioral data from the bees in the control treatment (which were almost exclusively foraging from horizontal flowers). To calculate estimated energetic parameters (means ± 95% CI) for the bees in the low-difference treatment assuming that they had continued foraging on the vertical surfaces (Figures 2C and 2E), we used the behavioral data from the high-difference-treatment bees (which were mostly foraging from the vertical flowers). Calculation of these alternative strategies is realistic as drinking speed does not change with concentration below concentrations of around 35–40% w/w.^54^

The RER and EE were compared for the first and final ten drinking visits between treatments using ANOVAs, with pairwise differences analyzed using Tukey post-hoc tests in cases where the ANOVA showed a significant difference between treatments (results, Figure S4). Comparisons between observed and estimated values for RER and EE for high- and low-difference treatments were performed using Welch’s t-tests (results). Residual plots were examined for all tests and data were log_10_-transformed as appropriate to meet test assumptions.

Mean handling time per drinking visit was calculated by averaging the time spent visiting and/or drinking from flowers (both horizontal and vertical) over every ten drinking visits for each bee (Figures 2G and S4D). This average included the time spent on any interaction with any flower over those ten drinking visits. The mean energy use per drinking visit was calculated using an equivalent method. The energy used during the time spent visiting and drinking from flowers (i.e., during all ‘handling time’ as defined above) was averaged over every ten drinking visits for each bee to give the mean energy use per drinking visit (Figures 2H and S4E). The specific calculations for energy used are described above in the section on ‘calculation of foraging energetics’. The mean time spent flying between flowers was calculated for each drinking visit by averaging the time spent flying (excluding any time spent visiting or drinking from flowers while hovering) over every ten drinking visits for each bee (Figure S4C). We compared the mean handling time, mean time spent flying between flowers and energy use per drink between bees in the high- and low-difference treatments using Welch’s t-tests (results, Figure S4). Residual plots were examined for all tests and data were log_10_-transformed as appropriate to meet test assumptions. All boxplots (Figures 2G, 2H, and S4C–S4E) were created using the boxplot() function in R, using the default value for range = 1.5. Selection of color schemes for figures was aided by using ColorBrewer.^63^

## Data Availability

• Data: The bee observation data file (CSV) and bee masses (CSV) have been deposited at Apollo, the institutional repository of the University of Cambridge, and are publicly available as of the date of publication. The DOI is listed in the key resources table. • Code: This paper does not report original code. • Any additional information required to reanalyze the data reported in this paper is available from the lead contact upon request.

## Footnotes

Supplemental information can be found online at https://doi.org/10.1016/j.isci.2023.108071.

## References

[1] Raine N.E., Chittka L. (2007). The adaptive significance of sensory bias in a foraging context: floral colour preferences in the bumblebee *Bombus terrestris*. PLoS One.

[2] Schiestl F.P., Dötterl S. (2012). The evolution of floral scent and olfactory preferences in pollinators: Coevolution or pre-existing bias?. Evolution.

[3] Whitney H.M., Chittka L., Bruce T.J.A., Glover B.J. (2009). Conical epidermal cells allow bees to grip flowers and increase foraging efficiency. Curr. Biol..

[4] Whittall J.B., Hodges S.A. (2007). Pollinator shifts drive increasingly long nectar spurs in columbine flowers. Nature.

[5] Vallejo-Marín M. (2019). Buzz pollination: studying bee vibrations on flowers. New Phytol..

[6] Bailes E.J., Ollerton J., Pattrick J.G., Glover B.J. (2015). How can an understanding of plant-pollinator interactions contribute to global food security?. Curr. Opin. Plant Biol..

[7] Solvi C., Gutierrez Al-Khudhairy S., Chittka L. (2020). Bumble bees display cross-modal object recognition between visual and tactile senses. Science.

[8] Charlton N.L., Houston A.I. (2010). What currency do bumble bees maximize?. PLoS One.

[9] Schmid-Hempel P., Kacelnik A., Houston A.I. (1985). Honeybees maximize efficiency by not filling their crop. Behav. Ecol. Sociobiol..

[10] Riffell J.A., Alarcón R., Abrell L., Davidowitz G., Bronstein J.L., Hildebrand J.G. (2008). Behavioral consequences of innate preferences and olfactory learning in hawkmoth-flower interactions. Proc. Natl. Acad. Sci. USA.

[11] Jones P.L., Ryan M.J., Chittka L. (2015). The influence of past experience with flower reward quality on social learning in bumblebees. Anim. Behav..

[12] Córdoba S.A., Cocucci A.A. (2011). Flower power: Its association with bee power and floral functional morphology in papilionate legumes. Ann. Bot..

[13] Alcorn K., Whitney H., Glover B. (2012). Flower movement increases pollinator preference for flowers with better grip. Funct. Ecol..

[14] Kay Q.O.N., Daoud H.S., Stirton C.H. (1981). Pigment distribution, light reflection and cell structure in petals. Bot. J. Linn. Soc..

[15] Lihoreau M., Chittka L., Raine N.E., Kudo G. (2011). Trade-off between travel distance and prioritization of high-reward sites in traplining bumblebees. Funct. Ecol..

[16] Muth F., Keasar T., Dornhaus A. (2015). Trading off short-term costs for long-term gains: How do bumblebees decide to learn morphologically complex flowers?. Anim. Behav..

[17] Gegear R.J., Manson J.S., Thomson J.D. (2007). Ecological context influences pollinator deterrence by alkaloids in floral nectar. Ecol. Lett..

[18] Wang M.Y., Ings T.C., Proulx M.J., Chittka L. (2013). Can bees simultaneously engage in adaptive foraging behaviour andattend to cryptic predators?. Anim. Behav..

[19] Jones E.I., Dornhaus A. (2011). Predation risk makes bees reject rewarding flowers and reduce foraging activity. Behav. Ecol. Sociobiol..

[20] Pyke G.H. (1980). Optimal Foraging in Bumblebees: Calculation of Net Rate of Energy Intake and Optimal Patch Choice. Theor. Popul. Biol..

[21] Hodges C.M. (1981). Optimal foraging in bumblebees: Hunting by expectation. Anim. Behav..

[22] Pyke G.H. (1979). Optimal foraging in bumblebees: Rule of movement between flowers within inflorescences. Anim. Behav..

[23] Best L.S., Bierzychudek P. (1982). Pollinator Foraging on Foxglove (Digitalis purpurea): A Test of a New Model. Evolution.

[24] Cartar R.V. (1992). Morphological senescence and longevity - an experiment relating wing wear and life-span in foraging wild bumble bees. J. Anim. Ecol..

[25] Cartar R.V., Dill L.M. (1990). Colony energy requirements affect the foraging currency of bumble bees. Behav. Ecol. Sociobiol..

[26] Pattrick J.G., Symington H.A., Federle W., Glover B.J. (2020). The mechanics of nectar offloading in the bumblebee *Bombus terrestris* and implications for optimal concentrations during nectar foraging. J. R. Soc. Interface.

[27] Bailes E.J., Pattrick J.G., Glover B.J. (2018). An analysis of the energetic reward offered by field bean (*Vicia faba*) flowers: nectar, pollen and operative force. Ecol. Evol..

[28] Pyke G.H. (1984). Optimal foraging theory: a critical review. Annu. Rev. Ecol. Syst..

[29] Houston A.I., Mcnamara J.M. (2014). Foraging currencies, metabolism and behavioural routines. J. Anim. Ecol..

[30] Balfour N.J., Shackleton K., Arscott N.A., Roll-Baldwin K., Bracuti A., Toselli G., Ratnieks F.L.W. (2021). Energetic efficiency of foraging mediates bee niche partitioning. Ecology.

[31] Wolf T.J., Ellington C.P., Begley I.S. (1999). Foraging costs in bumblebees: Field conditions cause large individual differences. Insectes Soc..

[32] Balfour N.J., Gandy S., Ratnieks F.L.W. (2015). Exploitative competition alters bee foraging and flower choice. Behav. Ecol. Sociobiol..

[33] Heinrich B. (1975). Thermoregulation in bumblebees II. Energetics of warm-up and free flight. J. Comp. Physiol. B.

[34] Kammer A.E., Heinrich B. (1974). Metabolic rates related to muscle activity in bumblebees. J. Exp. Biol..

[35] Combes S.A., Gagliardi S.F., Switzer C.M., Dillon M.E. (2020). Kinematic flexibility allows bumblebees to increase energetic efficiency when carrying heavy loads. Sci. Adv..

[36] Waldron F.A., Wiegmann D.D., Wiegmann D.A. (2005). Negative incentive contrast induces economic choice behavior by bumble bees. Int. J. Comp. Psychol..

[37] Nachev V., Thomson J.D., Winter Y. (2013). The psychophysics of sugar concentration discrimination and contrast evaluation in bumblebees. Anim. Cogn..

[38] Cnaani J., Thomson J.D., Papaj D.R. (2006). Flower choice and learning in foraging bumblebees: effects of variation in nectar volume and concentration. Ethology.

[39] Heinrich B. (2004).

[40] Kacelnik A., Houston A.I., Schmid-Hempel P. (1986). Central-place foraging in honey bees: the effect of travel time and nectar flow on crop filling. Behav. Ecol. Sociobiol..

[41] Wolf T.J., Schmid-Hempel P. (1989). Extra loads and foraging life span in honeybee workers. J. Anim. Ecol..

[42] Higginson A.D., Houston A.I. (2015). The influence of the food–predation trade-off on the foraging behaviour of central-place foragers. Behav. Ecol. Sociobiol..

[43] Seeley T.D. (1994). Honey bee foragers as sensory units of their colonies. Behav. Ecol. Sociobiol..

[44] Townsend-Mehler J.M., Dyer F.C., Maida K. (2011). Deciding when to explore and when to persist: A comparison of honeybees and bumblebees in their response to downshifts in reward. Behav. Ecol. Sociobiol..

[45] Kim W., Gilet T., Bush J.W.M. (2011). Optimal concentrations in nectar feeding. Proc. Natl. Acad. Sci. USA.

[46] Brown M., Brown M.J.F. (2020). Nectar preferences in male bumblebees. Insectes Soc..

[47] Bramlett J.L., Perdue B.M., Evans T.A., Beran M.J. (2012). Capuchin monkeys (Cebus apella) let lesser rewards pass them by to get better rewards. Anim. Cogn..

[48] Miller R., Frohnwieser A., Schiestl M., McCoy D.E., Gray R.D., Taylor A.H., Clayton N.S. (2020). Delayed gratification in New Caledonian crows and young children: influence of reward type and visibility. Anim. Cogn..

[49] Vanderveldt A., Oliveira L., Green L. (2016). Delay Discounting: Pigeon, Rat, Human – Does it Matter?. J. Exp. Psychol. Anim. Learn. Cogn..

[50] Koch K., Schulte A.J., Fischer A., Gorb S.N., Barthlott W. (2008). A fast, precise and low-cost replication technique for nano- and high-aspect-ratio structures of biological and artificial surfaces. Bioinspir. Biomim..

[51] Schulte A.J., Koch K., Spaeth M., Barthlott W. (2009). Biomimetic replicas: Transfer of complex architectures with different optical properties from plant surfaces onto technical materials. Acta Biomater..

[52] Goulson D., Hawson S.A., Stout J.C. (1998). Foraging bumblebees avoid flowers already visited by conspecifics or by other bumblebee species. Anim. Behav..

[53] Stout J.C., Goulson D., Allen J.A. (1998). Repellent scent-marking of flowers by a guild of foraging bumblebees (Bombus spp.). Behav. Ecol. Sociobiol..

[54] Harder L.D. (1986). Effects of nectar concentration and flower depth on flower handling efficiency of bumble bees. Oecologia.

[55] Prŷs-Jones O., Corbet S. (2011).

[56] Longinotti M.P., Corti H.R. (2008). Viscosity of concentrated sucrose and trehalose aqueous solutions including the supercooled regime. J. Phys. Chem. Ref. Data.

[57] Heinrich B., Vogt F.D. (1993). Abdominal Temperature Regulation by Arctic Bumblebees. Physiol. Zool..

[58] R Core Team (2022). https://www.R-project.org/.

[59] Bates D.W., Zimlichman E., Bolker B., Walker S. (2015). Fitting Linear mixed-effects models using lme4. BMJ Qual. Saf..

[60] Hartig F. (2022). DHARMa: residual diagnostics for hierarchical (multi-level / mixed) regression models. R package version 0.4.5. https://CRAN.R-project.org/package=DHARMa.

[61] Manly B. (2006).

[62] Pattrick J.G., Shepherd T., Hoppitt W., Plowman N.S., Willmer P. (2017). A dual function for 4-methoxybenzaldehyde in Petasites fragrans? Pollinator-attractant and ant-repellent. Arthropod. Plant. Interact..

[63] Brewer C.A. (2022). http://www.ColorBrewer.org.

