# Supplementary material for: Bumblebees negotiate a trade-off between nectar quality and floral biomechanics
Source: iScience. 2023 Oct 24;26(11):108071. doi: 10.1016/j.isci.2023.108071 (PMC10725025; doi:10.1016/j.isci.2023.108071)
Supplement: Document S1. Figures S1–S4 and Tables S1 and S2 [file mmc1.pdf]

iScience, Volume *26*

Supplemental information

Bumblebees negotiate a trade-off between nectar quality and floral biomechanics

Jonathan G. Pattrick, Hamish A. Symington, Walter Federle, and Beverley J. Glover

Supplemental Information

Sensitivity of conclusions to model parameters

We calculated the rate of energy return to the colony (RER) and energy efficiency (EE) by estimating the metabolic rate for bumblebees using published values[1,2]. RER is dominated by the energy content of the nectar (which is an order of magnitude higher than the energy used during a foraging trip) and by the time spent on the foraging trip (Main text, Eq 1), which we measured, and so is relatively insensitive to changes in the value for metabolic rate. Furthermore, there is good agreement among different studies for the metabolic rate for flight[1,3–5], giving confidence in the assertion of the dominance of energy content of nectar over metabolic energy used in our calculations of RER.

In contrast, EE is much more dependent on the values for metabolic rate (Main text, Eq 2). Consequently, our conclusion on RER over EE as the preferred energy currency of nectar-foraging bumblebees is sensitive to the ratio of the chosen values for the metabolic rate of bumblebees in flight and while landed. Here we use values originally from Heinrich, where the metabolic rate during flight is around an order of magnitude higher than that of a landed bee[1,2]. While, as noted above, there is good agreement across different studies for the metabolic rate for flight, it is possible that Heinrich's value for bumblebees at rest may be an underestimate, as it was measured from bumblebees that were not attempting to regulate their body temperature[1].

In order to consider the possible effects of thermoregulation on our results, we draw on a model from Pyke[6], which explores under which conditions for a foraging bumblebee the excess heat generated during inter-flower flight is enough to offset the heat loss while landed on a flower. In the test phase of our experiment, heat loss while landed on flowers will be greatest for the control bees, which were almost exclusively visiting the horizontal flowers, on which they land to drink. We therefore calculated for these bees the mean time spent during flight and landed over their final ten drinking visits during the test phase, while foraging on flowers in the flight arena. This gave a proportion *p* of time spent in flight of 0.3773. We used the second half of equation 5 from Pyke[6], to calculate the extra energetic expenditure for maintaining thorax temperature while foraging

$$\max\left\{\left[k'(T_{th} - T_A) - 251\frac{\text{cal}}{\text{g}\times\text{hr}}mp - 0.67 \times c(T_{th})\frac{\text{cal}}{\text{g}\times\text{hr}}m(1-p)\right], 0\right\}, [1]$$

where p = the proportion of time in flight = 0.3773, T_{th} = thorax temperature = 37 °C, T_A = lab temperature = 21 °C, m = 0.1757 g (the mean mass of the bumblebees in the control treatment), $c(T_{th}) = 29$ and k' = 1.464 calories hr^{-1}. We calculated k' using equation 4 in Pyke[6], SI Eq 2,

$$k' = 10^{0.72}\frac{\text{cal}}{\text{hr}} \times \left(\frac{m_{th}}{1\text{g}}\right)^{0.45}, [2]$$

where m_{th} is the mass of the thorax, where following Pyke[6] $m_{th} = m/3$. This gives an extra energetic expenditure of 4.65 calories hr^{-1}, which, converted into SI units gives 0.00541 J s^{-1} for maintaining thorax temperature over the foraging period. Assuming this energetic expenditure is solely during the time when the bee is landed (i.e. dividing by the proportion of foraging time while landed, (1 – *p*)) and then dividing by *m* to give a mass-specific metabolic rate, we have an extra 0.0494 J g^{-1} s^{-1} for maintaining thorax temperature during non-flight activities. Adding these thermoregulatory costs to Heinrich's estimate of 0.034 J g^{-1} s^{-1} gives a mass-specific metabolic rate while landed of 0.034 + 0.0494 = 0.0834 J g^{-1} s^{-1}. This would make metabolic expenditure during flight only around 5.2 times that while landed.

To estimate any effect this extra expenditure may have, we recalculated EE and RER after adjusting the metabolic rate for bees while landed in the foraging arena to 0.0834 J g^{-1} s^{-1}. This change to mass-specific metabolic rate did not affect our conclusions. Interestingly, Balfour *et al.*[5], using a different rationale, use a similar value for the metabolic rate of bees while foraging on flowers, of 0.077 J g^{-1} s^{-1}. Hence our models are robust to the potential impact of temperature regulation on the metabolic rate of the bees while landed. Indeed it is possible that bumblebees may not control thorax temperatures as precisely as in the model above[6,7], in which case a mass-specific metabolic rate of 0.0834 J g^{-1} s^{-1} would represent the potential upper extreme of the metabolic rate while landed.

Asymptotic behaviour of energetic parameters

The conclusions presented in this manuscript rely on the fact that the bees are at, or near to, asymptotes for the calculated energetic currencies, i.e. they are unable to optimise their foraging much further. Evidence that this is the case comes from comparing the RER and EE between treatments and across the familiarisation and test phases. The bees in the control treatment (foraging on 35% w/w sucrose) show asymptotic behaviour in the familiarisation phase for both RER and EE (fig. S4A,B). These asymptotes should also be the maximum RER/EE the bees in the control treatment can achieve during the test phase as they are foraging on the same sucrose concentration. Indeed, by the end of the test phase, the bees in the control treatment had reached the same asymptote, with no difference in RER or EE between the test or familiarisation phases for the final ten drinking visits (paired t-test, RER: t_{11} = 0.82, p = 0.43, EE: t_{11} = 0.21, p = 0.84).

For the test phase the bees in the low-difference treatment are mainly foraging from the horizontal surfaces (main text, Fig. 1E) containing 35% sucrose, and so their maximum possible RER and EE should be the same as the bees in the control treatment. There was no difference in RER or EE between the bees in the control treatment and the bees in the low-difference treatment for the final ten drinking visits of the test phase (Tukey, $q_{3,33}$ = 1.25, p = 0.65, $q_{3,33}$ = 1.62, p = 0.49 for RER and EE respectively, fig. S4A,B). Hence, the bees in the low-difference treatment also have an RER and EE near to/at the asymptotic value.

Whether the RER and EE of the bees in the high-difference treatment are approaching asymptotes is less clear. However, examination of the component factors of both RER and EE suggests these parameters are unlikely to increase further. Firstly, by the final ten choices of the test phase, there was no difference between treatments in the time spent flying in the arena (fig S4C, Kruskal-Wallis test, χ^2_2 = 2.6, p = 0.27). Furthermore, both handling time and energy use per flower are reasonably constant for bees in the high-difference treatment for the second half of the test phase (fig S4D and E, paired Wilcoxon test comparing the middle ten choices (choices 41-50) with the final ten choices, handling time: V = 50, n = 12, p = 0.42, energy use per flower: V = 55, n = 12, p = 0.23).

Finally, although some bees were much quicker, it took over 8 hours to take the slowest bee through training, familiarisation and test phases, hence the results presented here represent a substantial part of a day’s foraging for each bee.

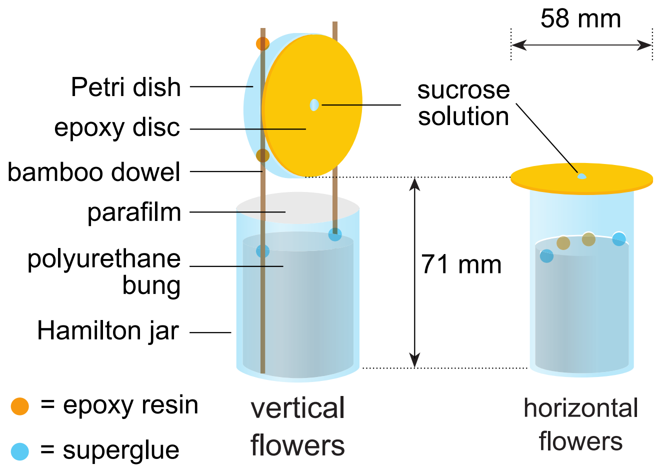

Figure S1. Design of the vertical and horizontal artificial flowers. Related to Figure 1A-B.

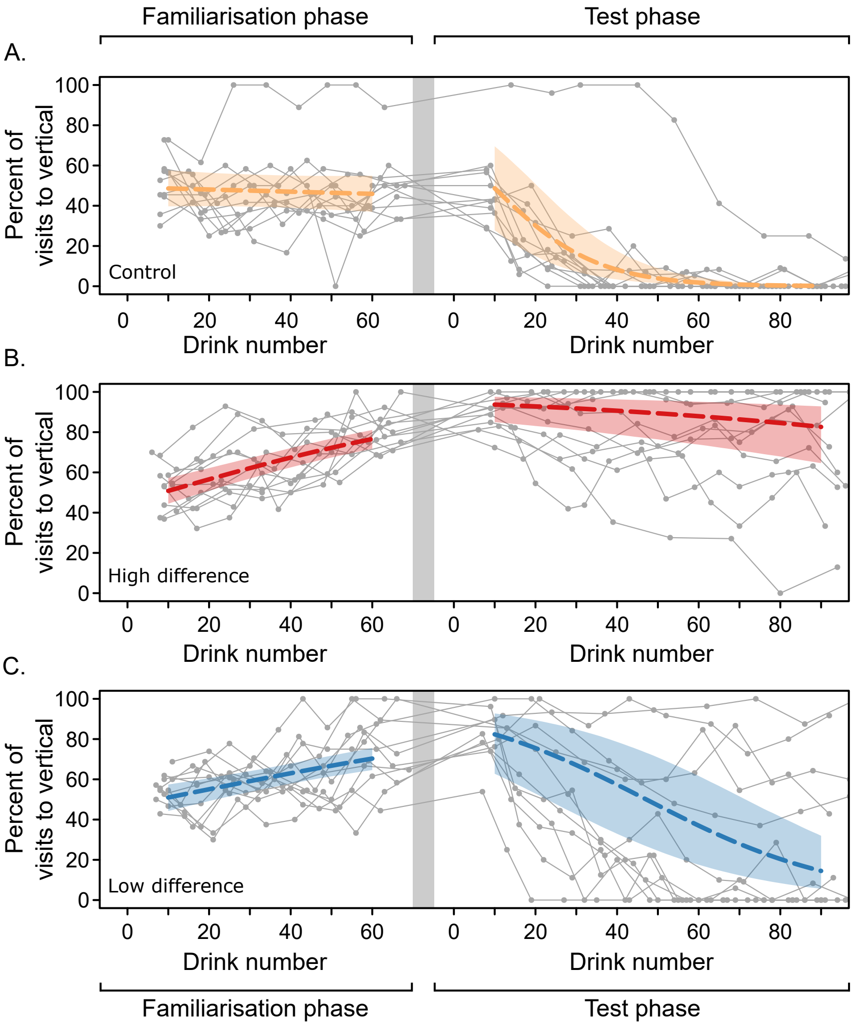

Figure S2. The proportion of all visits to vertical flowers for the familiarisation and test phases for the low-difference (**A**), high-difference (**B**) and control (**C**) treatments. Related to Figure 1C-E. The measured proportions at the end of each foraging trip for each individual bee are indicated by solid circles, plotted against the cumulative number of choices (drinking visits) made by the end of that foraging trip, with consecutive foraging trips for a bee connected by thin solid grey lines. Thick dashed lines are fitted models with 95 % CI bounds (shaded area). N = 12 bees per treatment. One useful comparison to note is that there was little difference in the proportion of visits to vertical flowers between high- and low-difference treatments by the end of the familiarization phase (MPMP [95% CI] at drink number 60: low-difference = 70.3% [64.6, 75.5%], high-difference = 76.6% [71.7, 81.1], $t_{15.1}$ = 0.97, p = 0.35), which suggests that the behavioural differences in the test phase between bees in these two treatments (see main text) are not simply the result of bees in the high-difference treatment learning to visit vertical flowers more effectively than bees in the low-difference treatment before the test phase.

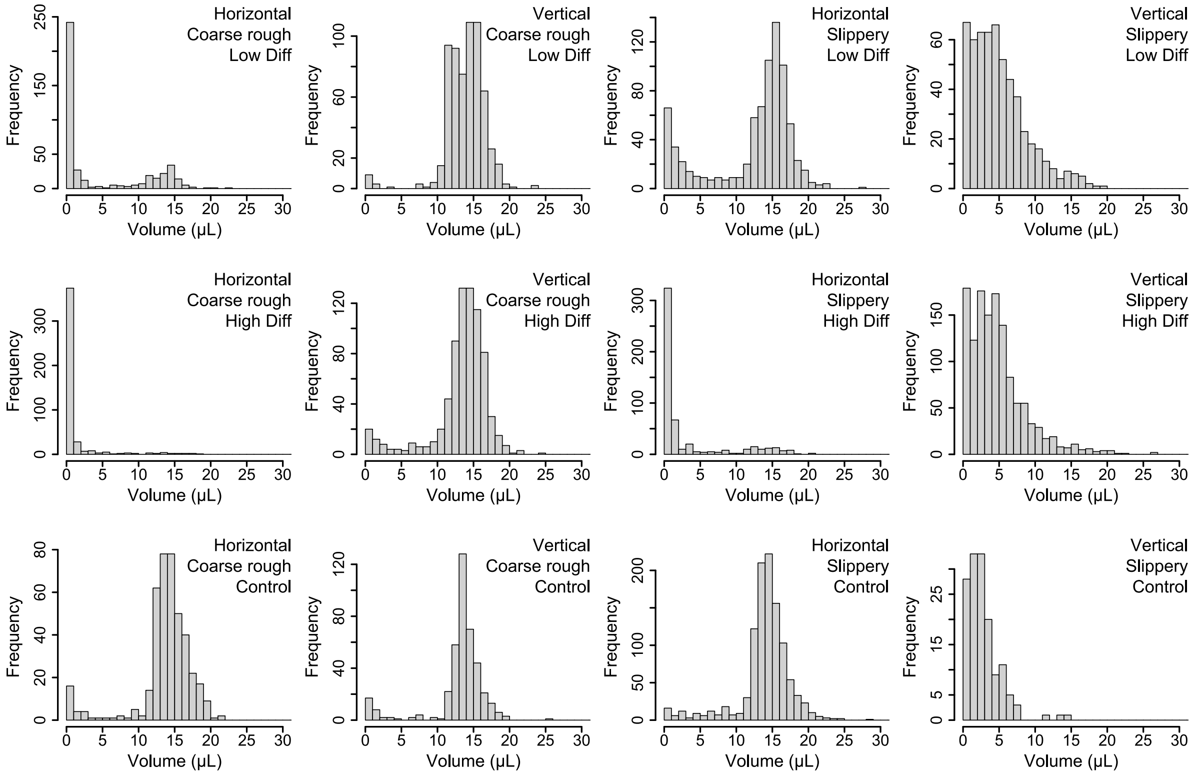

Figure S3. Histograms of the estimated volume of solution consumed for each flower visit where there was *any* contact between the bee's proboscis and the nectar, calculated for each treatment (low-difference, high-difference or control), surface type (coarse-rough or micro-rough (slippery)) and orientation (horizontal or vertical). Related to STAR Methods. Each bee was randomly assigned to a treatment and presented a choice between horizontal and vertical flowers first with coarse-rough surfaces (familiarisation phase), and then micro-rough surfaces (test phase). A preference for vertical or horizontal surfaces is apparent from the prevalence of visits with a low versus higher estimated volume. Bees had difficulty in drinking from vertical flowers with slippery micro-rough surfaces, and consequently there were few drinking visits where the bee managed to consume 15 µL in one go, even when this orientation was preferred. For calculation of foraging energetics, any volume resulting from a drinking time of 2 seconds or less was counted as a rejected visit and not used. Furthermore, as each flower was filled with 15 µL of solution, any estimates over this value were set to 15 µL. Note that y axes differ between plots.

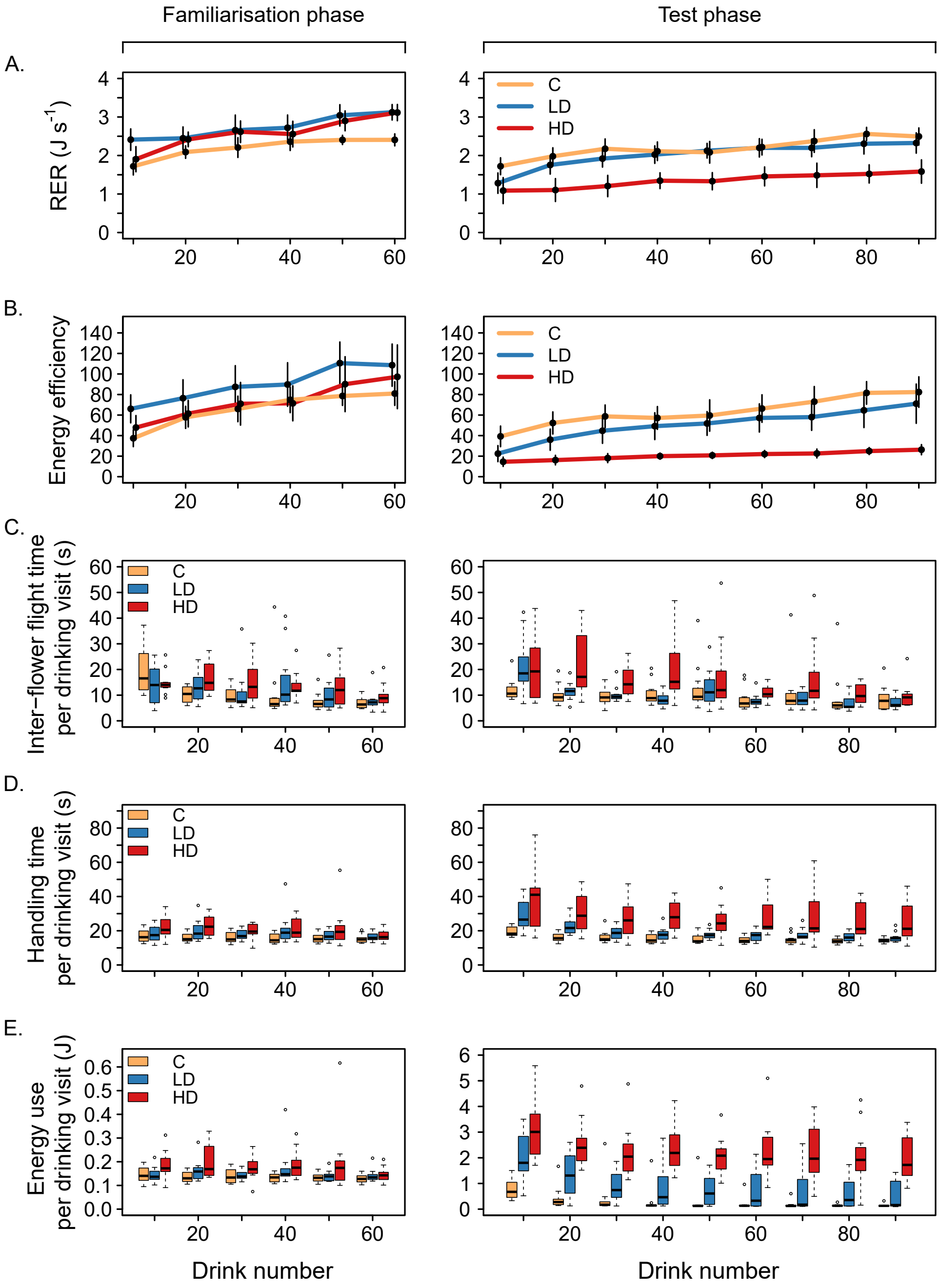

Figure S4. Foraging energetics for bees on the control (C, orange), low-difference (LD, blue) and high-difference (HD, red) treatments, over both the familiarisation and test phases, N = 12 bees per treatment. Related to Figure 2. **A.** Mean rate of energy return to the colony (RER) ±95% CIs, per foraging trip, averaged at every ten drinking visits. **B.** Mean energetic efficiency (EE) ±95% CIs per foraging trip, averaged at every ten drinking visits. During the test phase there were significant differences between treatments for the final ten choices for both RER and EE (ANOVA, $F_{2,33} = 12.8$, $p = 7.6 \times 10^{-5}$, $F_{2,33} = 22.8$, $p = 6.1 \times 10^{-7}$ respectively). RER and EE increase from the start to the end of the familiarisation and test phases for all treatments (Paired Wilcoxon tests of the first ten and final ten choices with Bonferroni corrections (for 12 tests), all $p < 0.05$). Points are offset to aid visibility in A and B. **C-E**, boxplots of mean times and energetic expenditure per drinking visit for different behaviours, averaged for every ten drinking visits. **C.** Mean times spent flying between flowers per drinking visit. In the test phase, there was no significant difference in flight time ($\log_{10}$-transformed) between low and high-difference treatment bees for either the first ten ($t_{20.9} = 0.36$, $p = 0.72$) or the final ten ($t_{20.9} = 1.65$, $p = 0.11$) drinks, suggesting this is not an important component to the difference between RER and EE between these two treatments. One extreme outlier (test phase, high-difference treatment, drink number 20, time = 118 s) is not visible on the plot. **D.** Boxplots of means of handling times per drinking visit **E.** Boxplots of means of the energy used on flower handling per drinking visit. N.B. the y-axis differs markedly in scale between the familiarisation and test phases for E.

Table S1. The mean (± SD) number of drinks per foraging trip over the first ten (start) and final ten (end) drinking visits for the different treatments. Related to STAR Methods.

Treatment	Familiarisation phase		Test phase	
	**Start**	**End**	**Start**	**End**
Control	8.9 ± 0.8	8.4 ± 0.9	9.2 ± 1.7	8.9 ± 1.1
Low-difference	8.8 ± 1.4	7.8 ± 1.1	9.8 ± 2.2	8.9 ± 2.0
High-difference	9.1 ± 1.6	8.8 ± 1.7	10.3 ± 1.8	8.9 ± 2.3

For the test phase, there was no evidence of any influence of treatment on the number of drinks per foraging trip, neither as an interaction with the number of completed drinking visits ($F_{2,33} = 0.84$, $p = 0.44$) nor as a main effect ($F_{2,33} = 0.35$, $p = 0.71$). For the familiarisation phase, there was also no effect of treatment on the number of drinks per foraging trip, neither as an interaction ($F_{2,33} = 1.31$, $p = 0.28$) nor as a main effect ($F_{2,33} = 0.92$, $p = 0.41$). For both test and familiarisation phases there was a small decrease in the number of drinks per foraging trip from the first ten drinking visits to the final ten drinking visits ($F_{1,33} = 7.57$, $p = 0.0096$ and $F_{1,33} = 11.3$, $p = 0.0020$, for test and familiarisation phases respectively). These data were modelled with linear mixed effects models using the R package nlme[8].

Table S2. Coefficients for the intercepts (back-transformed) and slopes (untransformed) of logistic regression models of the proportion of events (drinks or all visits) to vertical surfaces over consecutive drinks for the three treatments. Related to Figure 1C-E.

	**Familiarisation phase (coarse-rough surface)**		**Test phase (slippery surface)**	
Treatment / Parameter	Intercept	Slope	Intercept	Slope
**Drinks**				
Low difference (35% vs 50%)	0.63 (p = 0.14)	0.035 (p = 2.3 x 10^{-9})	0.71 (p = 0.19)	-0.032 (p < 2 x 10^{-16})
High difference (20% vs 50%)	0.71 (p = 0.043)	0.088 (p = 3.6 x 10^{-9})	1.00 (p = 5.1 x10^{-5})	-0.021 (p = 1.3 x 10^{-5}) †
Control (35% vs 35%)	0.54 (p = 0.51)	-0.0043 (p = 0.29)	0.16 (p = 0.065)	-0.077 (p = 2.4 x 10^{-11})
**All visits**				
Low difference (35% vs 50%)	0.47 (p = 0.41)	0.016 (p = 2.2 x 10^{-7})	0.88 (p = 0.00019)	-0.041 (p < 2 x 10^{-16})
High difference (20% vs 50%)	0.45 (p = 0.18)	0.023 (p = 3.2 x 10^{-15})	0.94 (p = 1.1 x 10^{-8})	-0.014 (p = 4.8 x 10^{-16}) †
Control (35% vs 35%)	0.49 (p = 0.86)	-0.0022 (p = 0.51)	0.68 (p = 0.11)	-0.079 (p < 2 x 10^{-16})

Note that the back-transformed intercept is directly interpretable as a proportion of choices to vertical, whereas the sign and magnitude of the untransformed slope indicates the degree to which the relationship is positive or negative. p-values indicate significant departure from 0.5 for the intercept or from 0 for the slope. †The two models (drinks and all visits) on the slippery surface for the large difference treatment used all the bees, including the two which switched to foraging mainly on the horizontal surfaces. On removing these bees from the model, the intercept (again back-transformed) and slope respectively are: 1.00 (p = 0.00062) and 0.0084 (p = 0.25) for drinks and 0.94 (p = 5.0 x10^{-8}) and -0.0042 (p = 0.048) for all visits. Consequently, although the bees in the high-difference treatment which continued to forage from the vertical surfaces did make some exploratory (non-feeding) visits to horizontal surfaces, there was no evidence that these bees were in the process of switching to foraging from horizontal flowers.

SI References

1. Kammer, A.E., and Heinrich, B. (1974). Metabolic rates related to muscle activity in bumblebees. J. Exp. Biol. *61*, 219–227.

2. Heinrich, B. (1975). Thermoregulation in bumblebees II. Energetics of warm-up and free flight. J. Comp. Physiol. *96*, 155–166. 10.1007/bf00706595.

3. Wolf, T.J., Ellington, C.P., and Begley, I.S. (1999). Foraging costs in bumblebees: Field conditions cause large individual differences. Insectes Soc. *46*, 291–295. 10.1007/s000400050148.

4. Combes, S.A., Gagliardi, S.F., Switzer, C.M., and Dillon, M.E. (2020). Kinematic flexibility allows bumblebees to increase energetic efficiency when carrying heavy loads. Sci. Adv. *6*. 10.1126/sciadv.aay3115.

5. Balfour, N.J., Gandy, S., and Ratnieks, F.L.W. (2015). Exploitative competition alters bee foraging and flower choice. Behav. Ecol. Sociobiol. *69*, 1731–1738. 10.1007/s00265-015-1985-y.

6. Pyke, G.H. (1980). Optimal foraging in bumblebees: Calculation of net rate of energy intake and optimal patch choice. Theor. Popul. Biol. *147*, 232–246.

7. Heinrich, B. (1972). Energetics of temperature regulation and foraging in a bumblebee, Bombus terricola kirby. J. Comp. Physiol. *77*, 49–64. 10.1007/BF00696519.

8. Pinheiro, J., Bates, D., DebRoy, S., Sarkar, D., and R Core Team. (2022). nlme: Linear and Nonlinear Mixed Effects Models. R package version 3.1-155. https://CRAN.R-project.org/package=nlme.
